# Mental health mobile apps for patients: Psychiatrists’ concerns

**DOI:** 10.1192/j.eurpsy.2021.928

**Published:** 2021-08-13

**Authors:** S. Hanft-Robert, K. Tabi, H. Gill, A. Endres, R.M. Krausz

**Affiliations:** 1 Department Of Medical Psychology, University Medical Center Hamburg-Eppendorf, Hamburg, Germany; 2 Institute Of Mental Health, Centre For Health Evaluation And Outcome Sciences, Department Of Psychiatry, Faculty of Medicine, University of British Columbia, Vancouver, Canada

**Keywords:** mobile apps, psychiatry, mental health, concerns

## Abstract

**Introduction:**

The use of mobile apps aimed at supporting patients with a mental illness is rapidly increasing.

**Objectives:**

The presented results explore psychiatrists’ concerns about mobile apps for patients with a mental illness. These results are part of a larger study that examines psychiatrists’ attitudes regarding the use and development of mobile apps.

**Methods:**

In the qualitative exploratory study, semi-structured interviews were conducted with 18 psychiatrists in Czech Republic, Austria, and Slovakia. Psychiatrists were recruited via snowball sampling. The interviews were digitally recorded, transcribed verbatim, translated into English, and content analyzed using deductive and inductive category development.

**Results:**

There were mixed feelings regarding mobile apps for patients with mental illness. While psychiatrists emphasized certain benefits (e.g. increasing patients’ treatment motivation and engagement), several concerns were also expressed, especially by psychiatrists who were generally unfamiliar with mobile apps. They feared being replaced; were afraid that patients would act as their own doctors, thereby damaging their health; stressed that mobile apps could not respond or be tailored to an individual the same way psychiatrists could tailor treatment to a patient.

**Conclusions:**

The psychiatrists who were more likely to have concerns about mental health apps were those who were generally unfamiliar with the apps and/or thought the apps aim to replace, rather than support, face-to-face treatment. Thus, clinicians and patients should be familiarized with the use of such mobile apps and educated on how they could support the face-to-face treatment.

